# Supplementary figures and images for: Epstein-barr virus latent membrane protein 1 targets cIAP1, cIAP2 and TRAF2 for proteasomal degradation to activate the non-canonical NF-κB pathway
Source: PLoS Pathog. 2026 Jan 26;22(1):e1013898. doi: 10.1371/journal.ppat.1013898 (PMC12872027; doi:10.1371/journal.ppat.1013898)

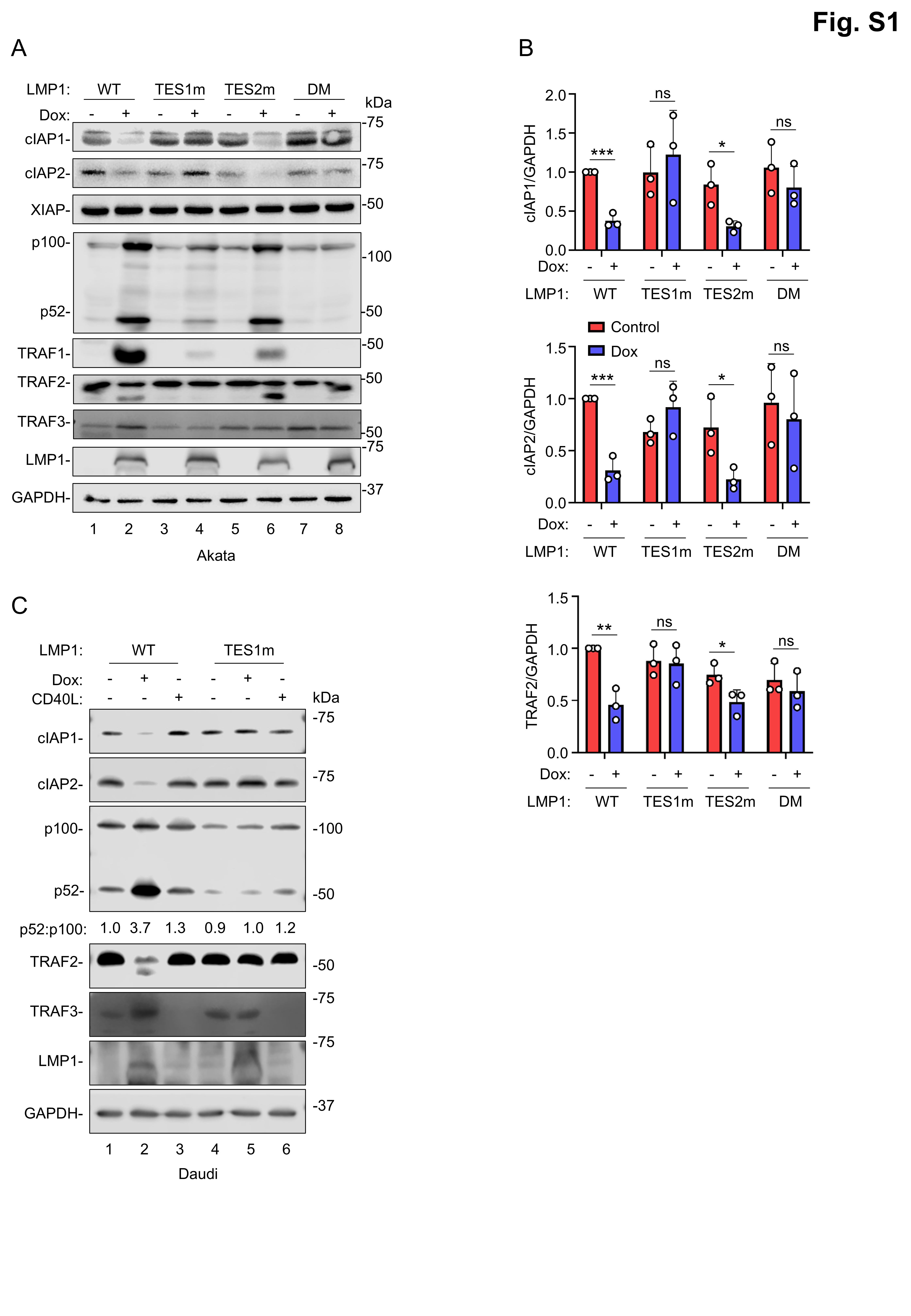

Supplement: S1 Fig — (A) Analysis of LMP1 TES1 vs TES2 signaling effects on cIAP1/2 and TRAF levels. Immunoblot analysis of WCL from Akata cells induced for WT, TES1m, TES2m or DM LMP1 expression by 250 ng/mL Dox for 24 hours. Blots are representative of n = 3 experiments. (B) Relative fold changes + SD of GAPDH load-controlled cIAP1, cIAP2 or TRAF2 values, based on densitometry from n = 3 replicates of immunoblots as shown in (A). Values in vehicle control treated cells uninduced for WT LMP1 expression were set to 1. (C) Analysis of LMP1 and CD40L effects on cIAP1, cIAP2, and TRAFs expression in Daudi Burkitt cells. Immunoblot analysis of whole cell lysates (WCL) from Cas9 + Daudi Burkitt B-cells induced for WT or TES1m LMP1 expression by addition of 250ng/mL doxycycline (Dox) for 24 hours or treated with 50 ng/mL CD40L for 1 hour. p52:p100 ratios are indicated. Statistical significance was assessed by two-tailed unpaired Student’s t-test (B). ns, not significant, *p < 0.05, **p < 0.01, ***p < 0.001. (TIF) [file ppat.1013898.s001.TIF]

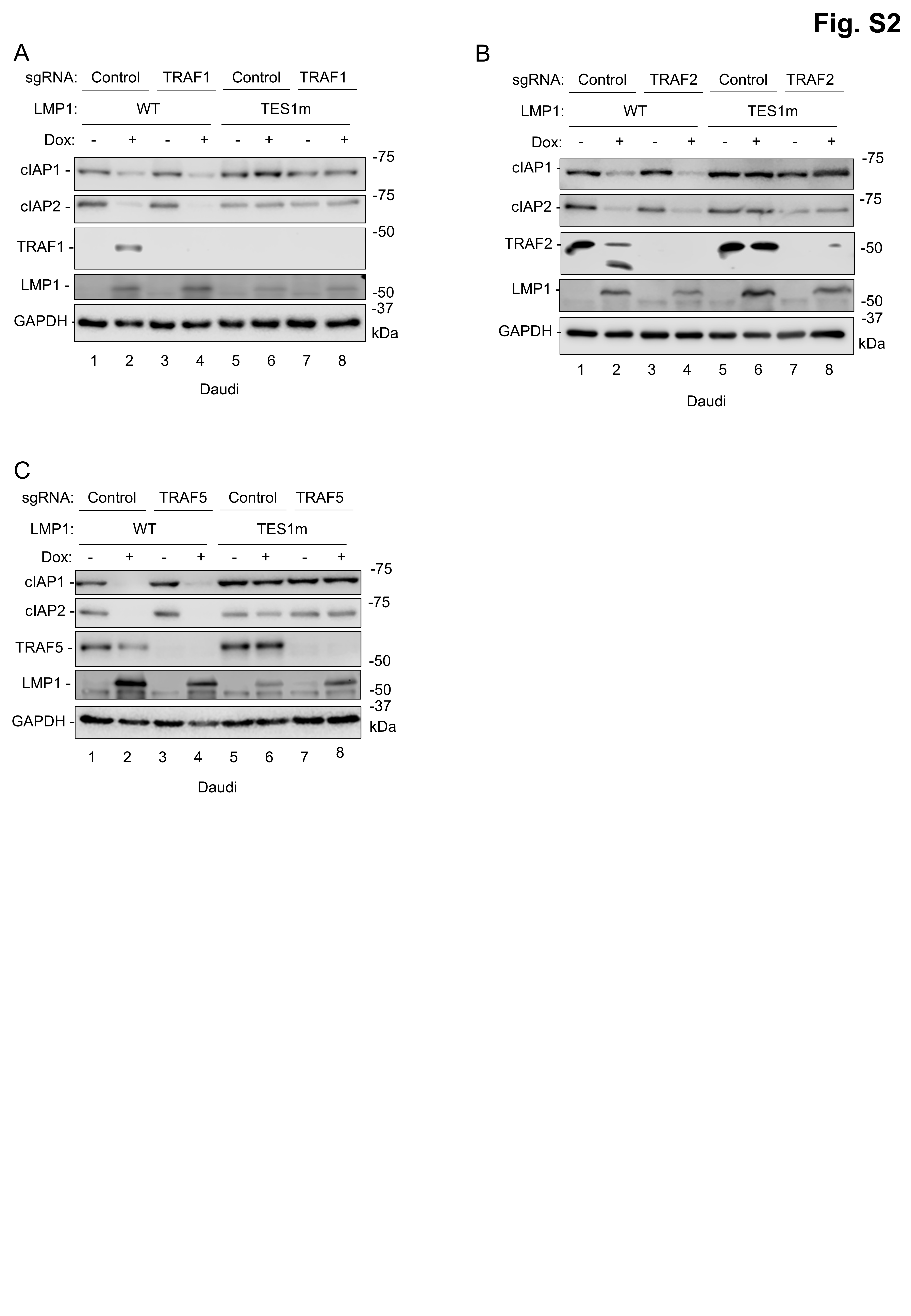

Supplement: S2 Fig — (A) Immunoblot analysis of WCL from Cas9 + Daudi cells that expressed control or TRAF1 targeting sgRNA and that were induced for LMP1 WT or TES1m expression by 250ng/mL Dox for 24 hours. (B) Immunoblot analysis of WCL from Cas9 + Daudi cells that expressed control or TRAF2 targeting sgRNA and that were induced for LMP1 WT or TES1m expression by 250ng/mL Dox for 24 hours. (C) Immunoblot analysis of WCL from Cas9 + Daudi cells that expressed control or TRAF5 targeting sgRNA and that were induced for LMP1 WT or TES1m expression by 250ng/mL Dox for 24 hours. Blots are representative of n = 3 experiments. (TIF) [file ppat.1013898.s002.TIF]

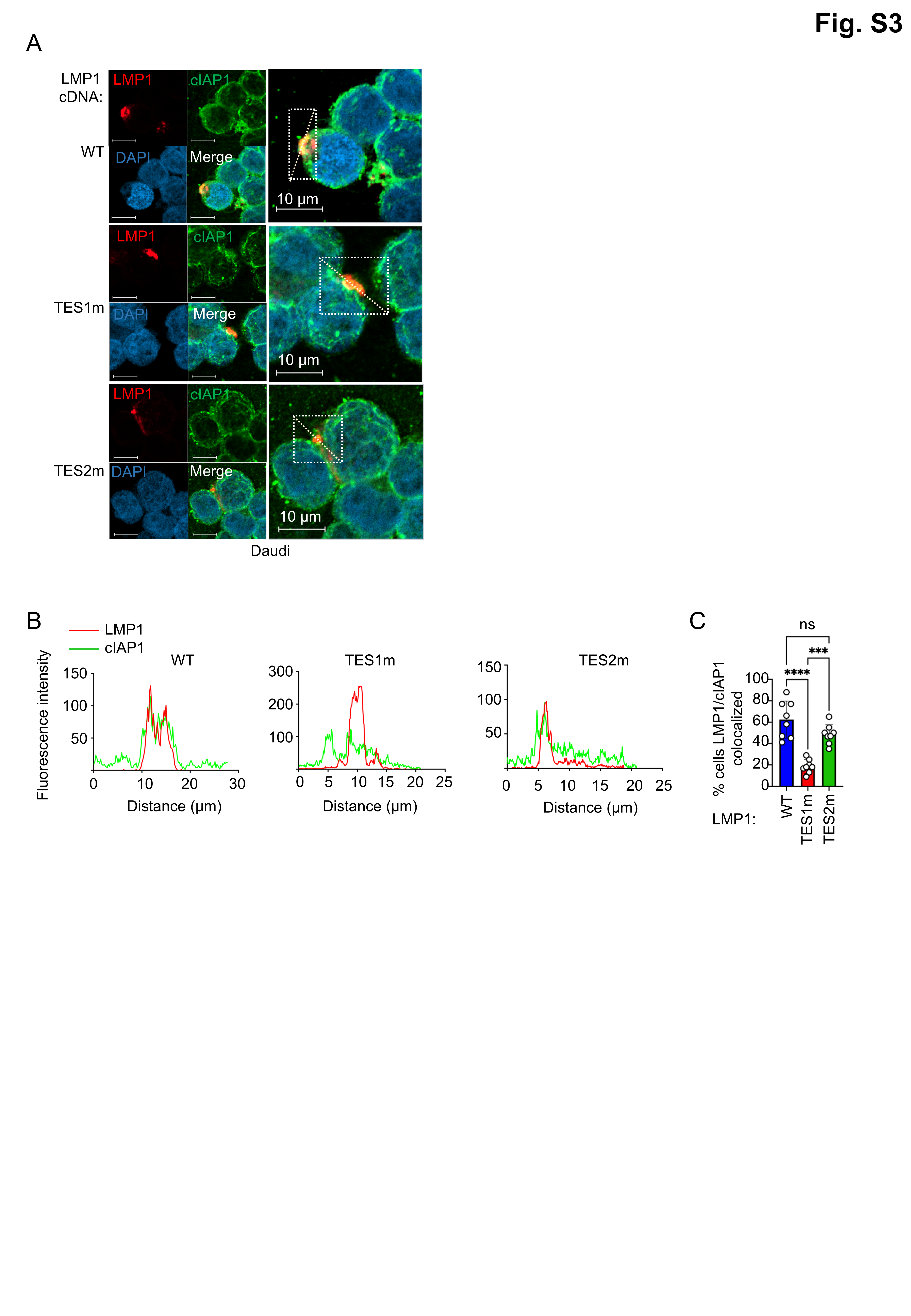

Supplement: S3 Fig — (A) Immunofluorescence analyses of cIAP1 and LMP1 localization in Daudi cells. WT, TES1m, TES2m or DM LMP1 expression was induced by 250ng/mL Dox for 24 hours, followed by treatment with 5 μM MG132 for 6 hours. Images are representative of 10 randomly chosen fields per sample. (B) Line scanning of cIAP1 (green) and LMP1 (red) fluorescence intensity within the annotated white rectangles shown in panel A. (C) Quantification of cells with overlapping cIAP1 and LMP1 signal in Daudi cells. Line scanning was performed with Zeiss Zen Lite (Blue) software. Statistical significance was assessed by one-way ANOVA followed by Tukey’s multiple comparisons test. ns, not significant, ***p < 0.001, ****p < 0.0001. (TIF) [file ppat.1013898.s003.TIF]

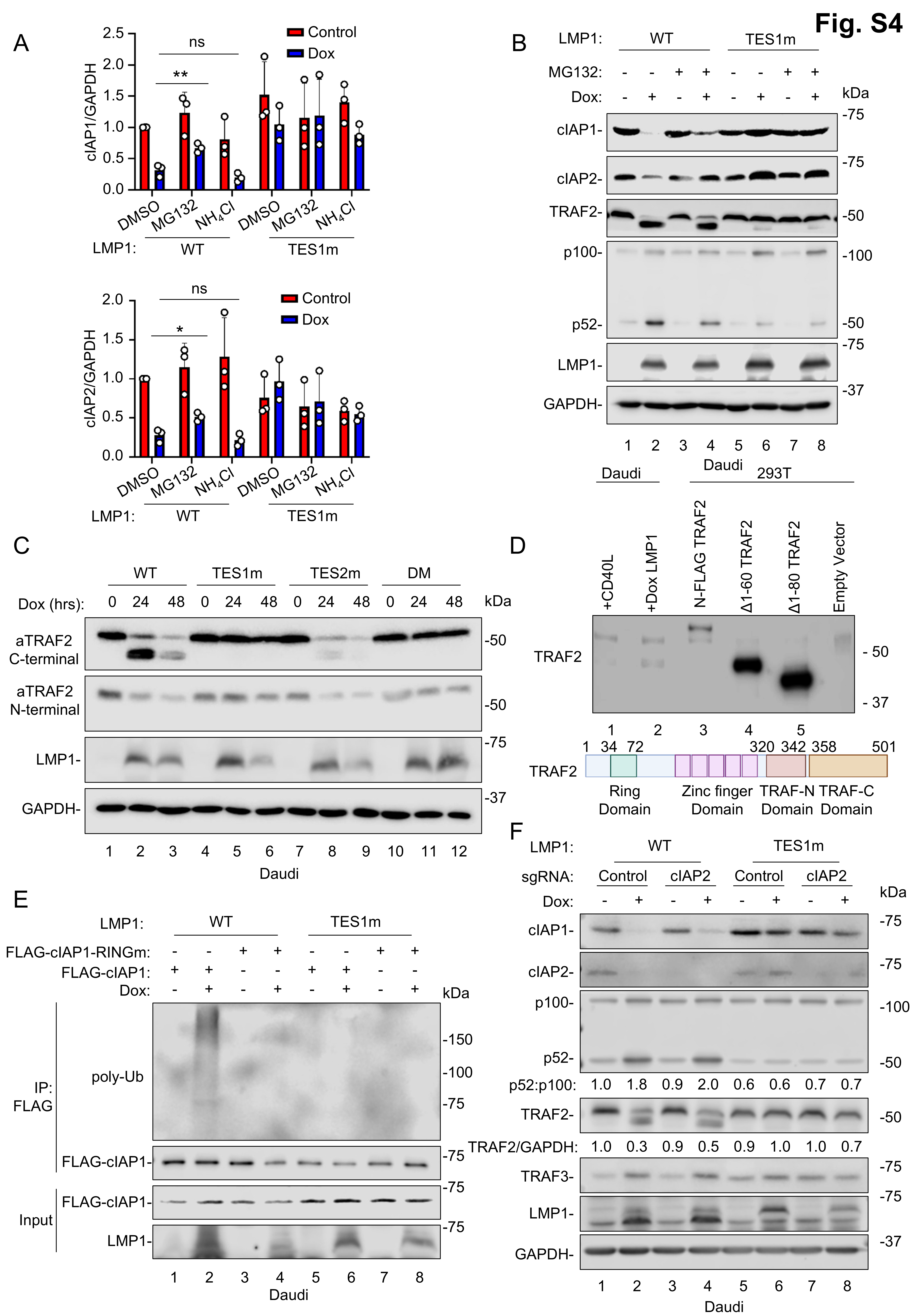

Supplement: S4 Fig — (A) Relative fold changes + SD of GAPDH-normalized cIAP1 or cIAP2 levels based on densitometry from n = 3 replicates as in Fig 5A. Values in vehicle control treated cells uninduced for WT LMP1 were set to 1. Statistical significance was assessed by two-tailed unpaired Student’s t test. ns, not significant, *p < 0.05, **p < 0.01. (B) Immunoblot analysis of WCL from Daudi cells induced for WT LMP1 expression by 250ng/mL Dox for 24 h, followed by treatment with 5μM MG132 for 8 hours. Blots are representative of n = 3 experiments. (C) Immunoblot analysis of WCL from Cas9 + Daudi Burkitt B-cells induced for WT, TES1m, TES2m, or DM LMP1 expression by addition of 250ng/mL Dox for 24 hours. TRAF2 blots were performed with antibodies raised against N‑terminal or C-terminal residues. (D) Immunoblot analysis of WCL from Daudi cells induced for WT LMP1 expression by 250ng/mL Dox for 24 h or from HEK293T cells overexpressing truncated TRAF2 constructs. Created in BioRender. Sun, Y. (2026) https://BioRender.com/yp7zxhj. (E) Immunoblot analysis of input versus anti-FLAG-cIAP1, immunopurified from Daudi cells that were transfected with expression vectors encoding FLAG-tagged cIAP1, cIAP1-RING mutant (RINGm, H588A) and empty vector as indicated. Daudi cells were induced for WT or TES1m LMP1 expression by 250 ng/mL Dox for 24 h. Cells were then treated with MG132 (5μM) or with NH4Cl (20mM) for an additional 8 hours before collection. Cell lysates were boiled and immunoprecipitated with anti-FLAG antibody and protein A/G magnetic beads. (F) Analysis of cIAP2 roles in LMP1 TES1-mediated effects on cIAP1, TRAFs and p52 expression. Immunoblot analysis of WCL from Cas9 + Daudi cells that expressed control vs cIAP2 targeting sgRNA and that were induced for LMP1 expression by Dox (250 ng/mL) for 24 hours. p52:p100 ratios are indicated. (TIF) [file ppat.1013898.s004.TIF]

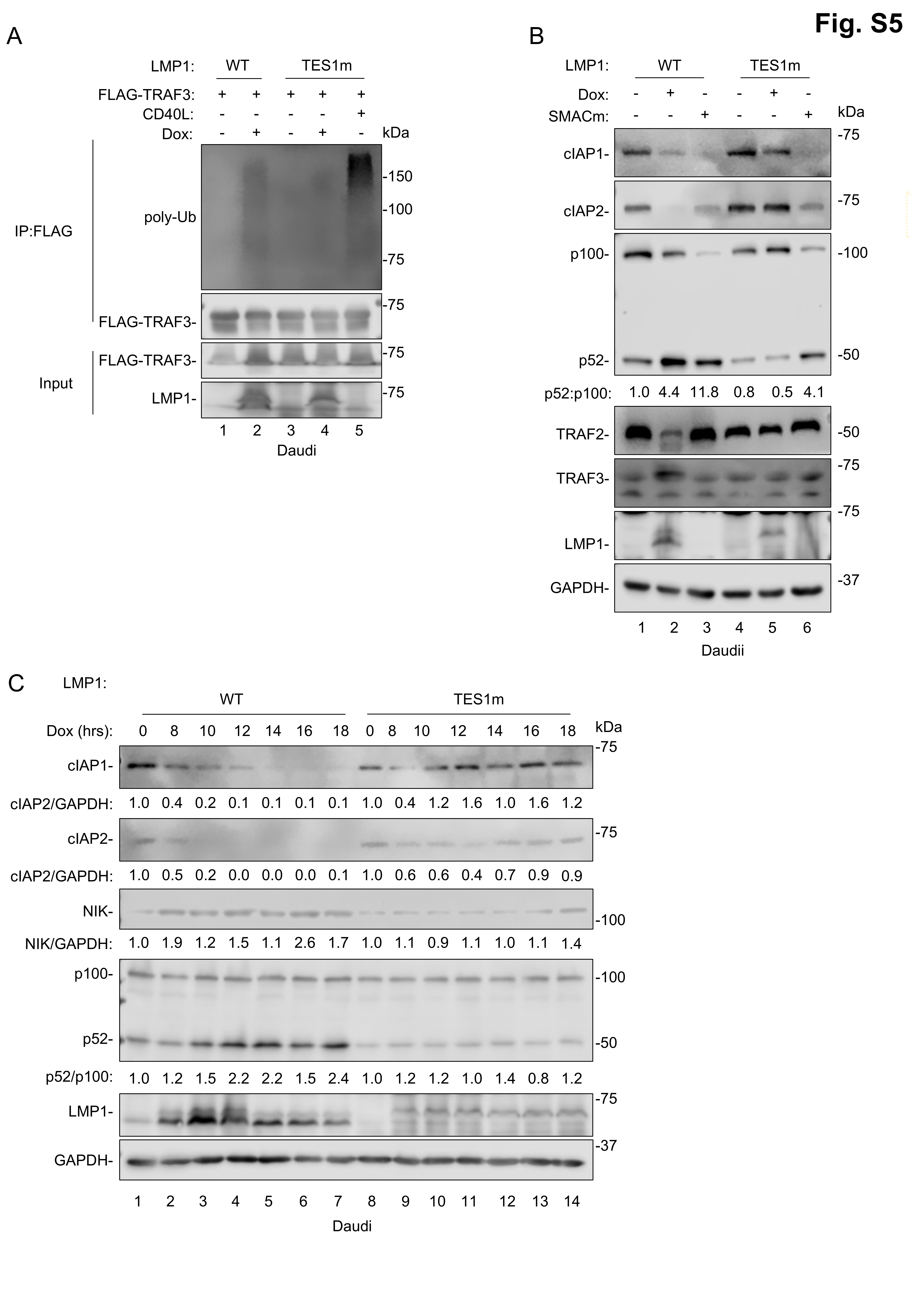

Supplement: S5 Fig — (A) Immunoblot analysis of TES1 vs CD40 driven TRAF3 polyubiquitination. Daudi cells were transfected with an expression vector encoding FLAG-tagged TRAF3 and induced for WT or TES1m LMP1 expression by Dox (250 ng/mL) for 24 h or instead stimulated by Mega-CD40L (50 ng/mL) for 1 hour. Cells were then treated with MG132 (5μM) for an additional 8 hours. Cell lysates were boiled to disrupt complexes and immunoprecipitated with anti-FLAG antibody and protein A/G magnetic beads. (B) Cross-comparison of LMP1 versus SMAC mimetic effects on cIAP1, cIAP2, TRAF2 and TRAF3 levels in B cells. Immunoblot analysis of WCL from Cas9 + Daudi Burkitt B-cells induced for WT or TES1m LMP1 expression by addition of 250ng/mL Dox for 24 hours, or treated with the SMAC mimetic (SMACm) birinapant (20 μM) for 8 hours. p52:p100 and TRAF2:GAPDH ratios are indicated. (C) Kinetic analysis of LMP1 TES1 signaling effects on non-canonical NF-κB pathway activation. Shown are immunoblot analysis of WCL from Daudi cells induced for WT or TES1m LMP1 expression by addition of Dox (250ng/mL) for the indicated hours. GAPDH or p100 normalized densitometry ratios are shown below each lane, presented as relative levels observed in unstimulated cells. (TIF) [file ppat.1013898.s005.TIF]
