# Supplementary material for: Epstein-barr virus latent membrane protein 1 targets cIAP1, cIAP2 and TRAF2 for proteasomal degradation to activate the non-canonical NF-κB pathway
Source: PLoS Pathog. 2026 Jan 26;22(1):e1013898. doi: 10.1371/journal.ppat.1013898 (PMC12872027; doi:10.1371/journal.ppat.1013898)
Supplement: Attachment — Submitted filename: Response to Reviewers.pdf [file ppat.1013898.s008.pdf]

Point by Point response.

We appreciate the editors and each of the reviewers' comments, each of which is addressed in the point-by-point response below. All regions that are changed in the manuscript are marked yellow. Our responses are marked with **">>Response:"**

Reviewers' Comments:

Reviewer's Responses to Questions

Part I - Summary
Please use this section to discuss strengths/weaknesses of study, novelty/significance, general execution and scholarship.

Reviewer #1: This is mechanistically detailed study on how EBV LMP1 TES1 activates the non-canonical NFκB pathway. The authors propose that LMP1, through TRAF3 recruitment, induces cIAP1 autoubiquitination and proteasomal degradation of cIAP1, cIAP2, and TRAF2, leading to p100→p52 processing. This mechanism is positioned as distinct from CD40/BAFF signaling. The study adds mechanistic detail to LMP1 signaling that has remained incompletely understood. The findings are solid, and conclusions are promising.

>>Response: We appreciate the reviewer's summary and comments, and address each of the specific points below.

Reviewer #2: This manuscript investigates how the EBV-encoded latent membrane protein-1 activates the non-canonical NF-κB pathway. The authors provide compelling evidence that TES1 of LMP1 associates with TRAF3 to recruit cIAP1/2 and TRAF2, leading to their ubiquitination and proteasomal degradation. This process results in NIK stabilisation and downstream p100 to p52 processing. The work identifies a novel mechanism of viral manipulation of host signalling and highlights potential therapeutic targets.

The study is significant and timely. The experiments are carefully designed and well controlled. However, a few mechanistic gaps, translational validations, and presentation issues should be addressed before publication.

>>Response: We appreciate the reviewer's summary and comments, and address each of the specific points below.

Reviewer #3: This manuscript by Sun, et al. addresses an important mechanistic question in EBV biology: how does LMP1 TES1 activate non-canonical NFκB signaling? The authors demonstrate that LMP1 TES1 recruits TRAF3, which serves as an essential adapter to bring cIAP1/2 ubiquitin ligases into proximity with LMP1, triggering their RING-dependent autoubiquitination and proteasomal degradation along with TRAF2. This degradation of negative regulators liberates NIK from constitutive degradation, enabling p100-to p52 processing and non-canonical NFκB activation.

The key finding that distinguishes this mechanism from CD40 and BAFF receptor signaling is that LMP1 preserves TRAF3 while targeting cIAP1/2 for degradation, whereas other immunoreceptors target TRAF3 itself. This resolves longstanding questions about how LMP1 activates non-canonical NFκB while requiring TRAF3, which was established as essential for LMP1 signaling by Xie et al in 2004. The work is well-controlled and uses appropriate genetic approaches including TRAF3 knockout B cells and cIAP1 RING domain mutants to demonstrate the mechanism. Overall, this represents a significant advance in understanding how this important viral oncogene usurps host signaling machinery.

<u>>>Response:</u> We appreciate the reviewer's summary and comments, and address each of the specific points below.

Part II – Major Issues: Key Experiments Required for Acceptance
Please use this section to detail the key new experiments or modifications of existing experiments that should be <u>absolutely</u> required to validate study conclusions.

Generally, there should be no more than 3 such required experiments or major modifications for a "Major Revision" recommendation. If more than 3 experiments are necessary to validate the study conclusions, then you are encouraged to recommend "Reject".

Reviewer #1: 1. The study rests on the premise that LMP1-mediated degradation of cIAP1/2 releases NIK from suppression. However, direct evidence of NIK stabilization, phosphorylation, or kinase activity is not shown in the setting.

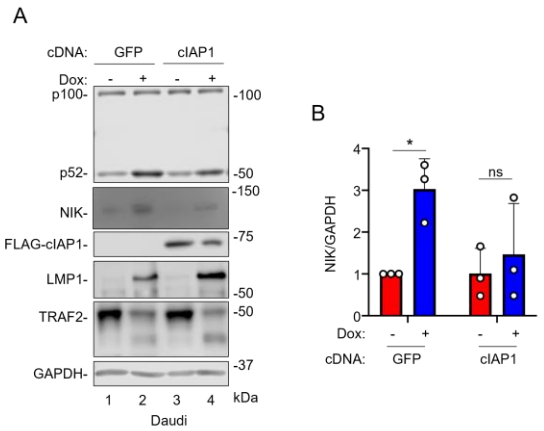

New Fig. 7. Effect of cIAP1 over-expression on LMP1-induced p100 processing and NIK stabilization. A) Immunoblot analysis of WCL from Daudi cells transfected with control GFP versus cIAP1 expression vectors for 24 hours and then mock induced or induced for LMP1 expression by Dox (250 ng/mL) for 24 hours, as indicated. Blots are representative of n=3 experiments. B) Quantification of NIK:GAPDH ratio from n=3 immunoblots as in (A). Shown are relative NIK:GAPDH ratios + SD from n=3 replicates. Values in vehicle control treated GFP expressing cells were set to 1. Statistical significance was assessed by two-way ANOVA followed by Tukey's multiple comparisons test. *p<0.05.

<u>>>Response:</u> We added NIK staining and quantification in new Fig. 7A-B. As shown in the figure, Daudi LMP1 expression results in NIK accumulation, and this could be suppressed by concurrent

cIAP1 overexpression. These results suggest that LMP1-induced cIAP degradation supports NIK stabilization and downstream p100:p52 processing, which is a downstream readout of NIK kinase activity.

2. The data suggest cIAP1 ligase activity is dominant, mediating degradation of both cIAP1 and cIAP2. Yet, the precise contribution of cIAP2 remains less clear. Selective depletion or complementation experiments could clarify whether cIAP2 is redundant, partially redundant, or required in specific contexts.

>>Response: As recommended, we expressed control or cIAP2 sgRNAs in Cas9+ Daudi cells with conditional WT or TES1m LMP1 expression. We then induced LMP1 expression for 24 hours and performed western blot analysis to assess whether cIAP2 is required for LMP1-mediated activation of non-canonical NF-κB signaling. As shown in the new Fig. S4F and below, cIAP2 knockout did not alter LMP1-mediated cIAP1 depletion, TRAF2 processing or on p100:p52 processing, indicating that cIAP2 is dispensable for LMP1-mediated activation of non-canonical NF-κB signaling.

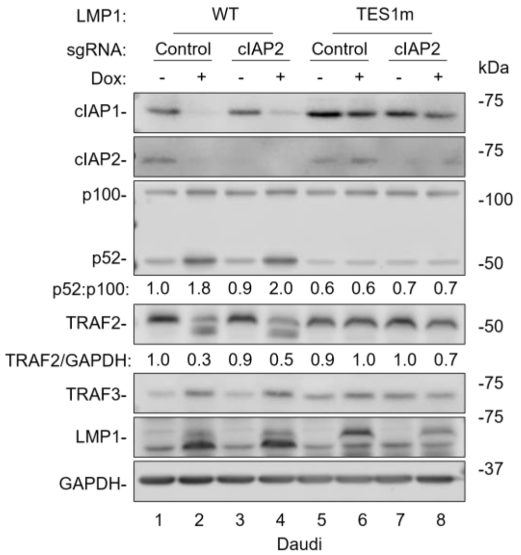

New Fig. S4F. cIAP2 roles in LMP1 TES1 signaling. Immunoblot analysis of WCL from Cas9+ Daudi cells that expressed control vs cIAP2 targeting sgRNA and that were induced for expression of WT or TES1m LMP1 by Dox (250 ng/mL) for 24 hours. p52:p100 and TRAF2:GAPDH ratios are indicated.

3. The appearance of a lower molecular weight TRAF2 species in Fig 1B, Fig2A, and S1A suggests partial cleavage in addition to degradation. The responsible protease(s) and its role in NFκB activation are not addressed.

>>Response: Thanks for this point. We do suspect that this represents LMP1 induced TRAF2 proteolytic cleavage. As TRAF1 has been reported to undergo caspase-mediated truncation (PMID: 11098060), we tested whether caspase activity was necessary for LMP1-driven TRAF2 processing. We treated cells with the pan-caspase inhibitor zVAD-fmk, with the proteasome inhibitor MG132 or the lysosome acidification inhibitor NH4Cl. However, as shown below (Fig. A below), none of these treatments eliminated LMP1 driven appearance of the TRAF2 low mol wt species, suggesting that other cellular proteases are responsible. To then characterize the low mol wt TRAF2 species to gain insights into the cleaved TRAF2 region and therefore possible proteases, we blotted with antibodies against epitopes in either the TRAF2 N- or C- termini. Only the C-terminal specific TRAF2 antibody

detected the truncated species (new Fig. S4C and Fig. B below), indicating that TES1 signaling stimulates cleavage and degradation of a TRAF2 region that includes the N-terminus. Moreover, comparison with engineered TRAF2 truncations revealed that the size of the fragment closely matches Δ1–60 TRAF2 (Fig. C below and new Fig. S4D), suggesting cleavage within or potentially adjacent to the RING domain (see panels C-D below). Because the RING domain is essential for TRAF2's E3 ligase activity and NFκB activation, removal of this segment would be expected to generate a fragment that retains receptor binding via the TRAF-C domain but that lacks ligase activity. We now discuss the potential functional consequence of this truncated TRAF2 species, which may impair TRAF2's ability to suppress NIK from within the TRAF2:TRAF3:cIAP1:cIAP2 complex. Inspection of TRAF2 sequence near residue 60 did not reveal obvious protease candidates. While we agree with the reviewer that it will be important to identify the TRAF2 protease, we look forward to doing so in a future study, but feel that the open ended search for it is beyond the scope of this study.

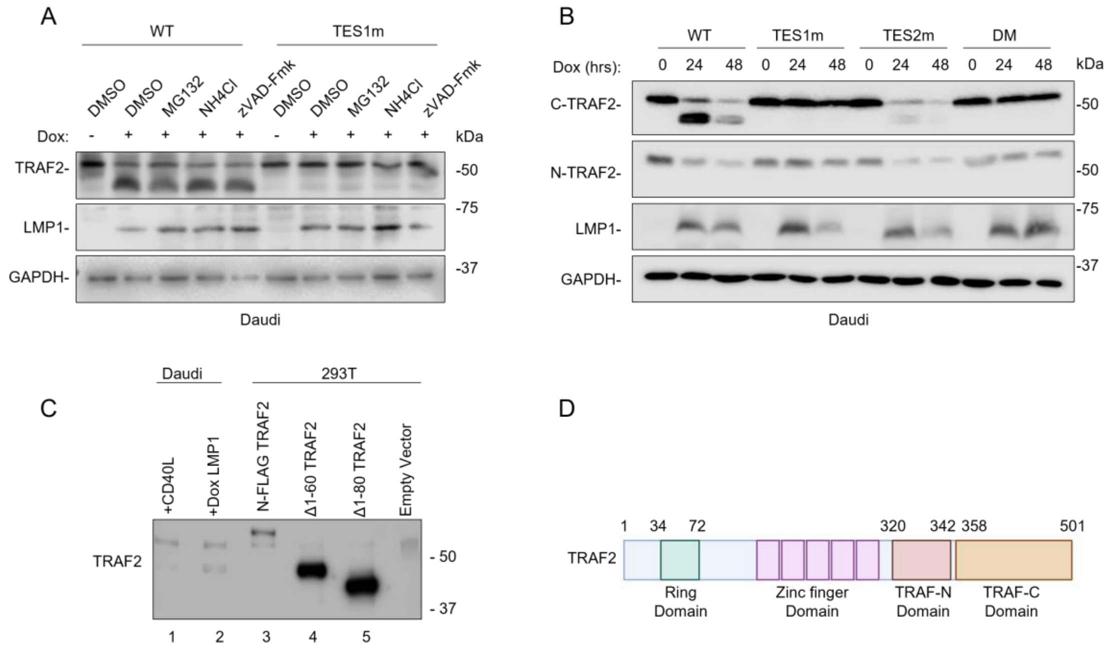

(A) Immunoblot analysis of WCL from Daudi cells induced for WT LMP1 expression by 250ng/mL Dox for 24 h, followed by treatment with 5µM MG132, 20 mM NH4Cl, or 20 uM zVAD-Fmk for 8 hours. (B) Immunoblot analysis of WCL from Cas9+ Daudi Burkitt B-cells induced for WT, TES1m, TES2m, or DM LMP1 expression by addition of 250ng/mL Dox for 24 hours. C-terminus-containing TRAF2 species were detected using anti-TRAF2 antibody (Proteintech #26846-1-AP) recognizing amino acids 438–501. N-terminal TRAF2 containing species were detected by anti-TRAF2 antibody (Invitrogen #MA5-41186) raised against an N-terminal peptide. (C) Immunoblot analysis of WCL from Daudi cells treated with CD40L or induced for WT LMP1 expression by 250ng/mL Dox for 24 h or from HEK293T cells overexpressing the indicated truncated TRAF2 constructs or empty vector. (D) Schematic representation of TRAF2 domains.

Reviewer #2: 1) The manuscript reports a lower molecular weight TRAF2 species upon LMP1 expression, yet the mechanism and functional significance of this remains unclear. Does this represent proteolytic cleavage?

>>Response: Please see response to reviewer #1 point 3 above.

2) The majority of data are derived from Burkitt lymphoma cell lines, LCLs, and 293T overexpression assays. To enhance translational impact, the authors should, if feasible, test whether cIAP1/2 downregulation is detectable in EBV-positive tumour samples (e.g., Hodgkin lymphoma or NPC biopsies). Even limited immunohistochemical or immunoblot evidence would add significant weight.

<u>>>Response:</u> We agree that this would be an exciting addition, but this is very difficult to achieve. Hodgkin Reed Sternberg cells are rare in tumors, and NPC is rare at our institution (many NPC occur in southern China, but tumors are not allowed to leave the country). LCLs are an important EBV model system that are thought to closely approximate immunoblastic lymphomas, in particular PTLD. We also wish to point out that cIAP1 and cIAP2 are known NF-κB transcriptional targets. Since LMP1 constitutively activates NF-κB, both cIAP1 and cIAP2 are transcriptionally upregulated in LMP1-positive cells. As a result, although LMP1 expression shortens the half-life of cIAP1 and cIAP2, we suspect that they remain detectable at steady state, as they are destabilized by LMP1 but also rapidly re-synthesized, a phenomenon we observe in LCLs (please see new Fig. 2A and 3C). Thus, immunohistochemistry of EBV+ Hodgkin or NPC tumor tissues would not necessarily reveal detectable downregulation of cIAP1 or cIAP2. Instead, we would need to perform pulse/chase analyses, which would require live Hodgkin Reed-Sternberg or NPC cells, which are not available. We wish to point out an analogy – both p100 and IkBa can be detected in LCLs, because both are destabilized by LMP1 signaling but also rapidly resynthesized as NF-kB targets.

3) Several ubiquitination experiments rely on HEK293T overexpression, which may not reflect physiological conditions. It would be helpful to confirm key findings (e.g., cIAP1 autoubiquitination) in EBV-positive B cells or LCLs to exclude cell-type specific artifacts.

<u>>>Response:</u> We tested cIAP1 or RING-mutant cIAP1 (cIAP1-RINGm) in Daudi cells. Consistent with our HEK293T results, LMP1 expression in EBV+ Daudi cells induced strong ubiquitination of FLAG-cIAP1, but failed to induce polyubiquitination of RING-mutant FLAG-cIAP1 (new Fig. S4E and below), suggesting that LMP1 activates the autoubiquitination activity of cIAP1 in the B-cell

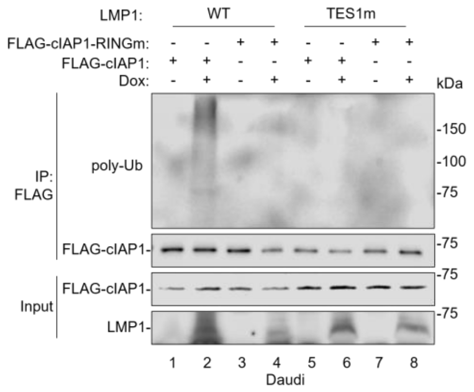

New Fig. S4E. Immunoblot analysis of input versus anti-FLAG-cIAP1, immunopurified from Daudi cells with expression of FLAG-tagged cIAP1, cIAP1-RING mutant (RINGm, H588A) or with empty vector control, as indicated. Daudi cells were induced for WT or TES1m LMP1 expression by 250 ng/mL Dox for 24 h. Cells were then treated with MG132 (5µM) for an additional 8 hours before collection. Cell lysates were boiled and immunoprecipitated with anti-FLAG antibody and protein A/G magnetic beads.

context.

4) The discussion emphasizes similarity to SMAC mimetics but does not experimentally test this concept. Including proof-of-principle data using SMAC mimetics (or other cIAP antagonists) in LCLs would bridge the mechanistic insights with therapeutic relevance.

>>Response: Thanks, we now cross compare SMAC mimetics and LMP1. To address this point, we directly cross-compared LMP1 and SMAC mimetic (SMACm) effects in Daudi cells. We chose to use Daudi cells with inducible LMP1 expression rather than LCLs, because LCLs are dependent on constitutive LMP1 expression for survival, and it would not be possible to cross-compare LMP1 with SMAC mimetic effects. Adding SMAC mimetics to LCL would combine LCL and SMAC mimetic effects, and we would therefore only be able to test superimposed LMP1 and SMACm effects, which might be difficult to interpret. As shown in new Fig. S5B and below, LMP1 expression or SMACm treatment each deplete cIAP1 and cIAP2, and each induced p100:p52 processing. However, SMAC mimetic birinapant treatment did not alter TRAF2 expression or electrophoretic mobility.

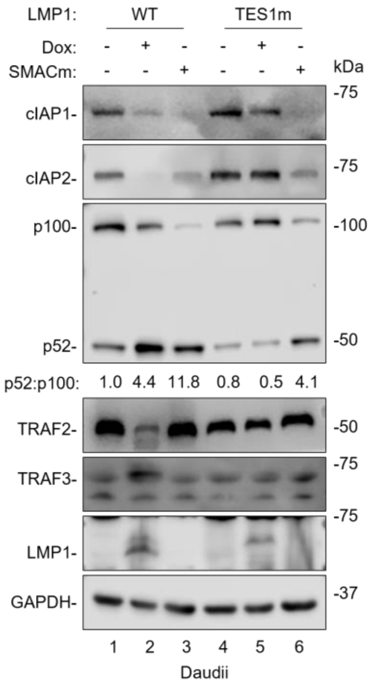

New Fig. S5B. Cross-comparison of LMP1 versus SMAC mimetic effects on cIAP1, cIAP2, TRAF2 and TRAF3 expression in Daudi Burkitt cells. Immunoblot analysis of whole cell lysates (WCL) from Daudi cells induced for WT or TES1m LMP1 expression by addition of 250ng/mL Dox for 24 hours, or treated with 20 uM SMACm birinapant for 8 hours. p52:p100 ratios are indicated.

Reviewer #3: 1. Lack of mechanistic detail regarding TRAF3-mediated cIAP1 activation. The manuscript establishes that TRAF3 is essential for cIAP1 recruitment and subsequent autoubiquitination but doesn't fully explain how TRAF3 binding activates cIAP1's E3 ligase activity. What is the mechanism by which TRAF3 association with LMP1 triggers cIAP1 RING domain activation? Is this through proximity-induced dimerization, conformational changes, or relief of autoinhibition?

<u>>>Response:</u> Thanks for the opportunity to address this interesting point. We agree that it would be of significant interest to define the mechanism by which LMP1 associated TRAF3 triggers cIAP1 activation. However, it is difficult to address this question experimentally, as LMP1 forms large oligomeric complexes (i.e. PMID: 38996527 and other refs). We are not aware of an assay to test whether or how high molecular weight LMP1/TRAF3 oligomers trigger proximity-induced cIAP1 dimerization or confirmational changes short of attempting a crystal structure. This would be highly challenging – the LMP1 C-terminal tail very likely contains large unstructured regions, and this is likely why it was not included in the recent atomic structure of LMP1 that instead focused on the TM regions.

We also wish to point out that cIAP1/2 are constitutively active in resting cells, and their activity is a critical brake on the NF-kB pathway by targeting NIK for degradation. Our results suggest that LMP1 and TRAF3 instead cause cIAP1 autodegradation, in a manner that may be analogous to the activity of SMAC mimetics. We speculate that LMP1/TRAF3 oligomers increase the intermolecular interactions amongst cIAP1 to trigger its autodegradation. By contrast, CD40 forms trimers upon CD40L engagement (PMID: 33091428), resulting in a more limited signaling platform. We now discuss this in the manuscript.

2. The paper should more explicitly address why TRAF3 remains stable while serving as an adapter for cIAP1/2 degradation, particularly given that cIAPs normally ubiquitinate TRAF3 in other signaling contexts. What protects TRAF3 from degradation in LMP1 complexes? The authors should analyze TRAF3 ubiquitination patterns in LMP1-expressing cells and consider whether LMP1 induces post-translational modifications that alter TRAF3's susceptibility to cIAP-mediated degradation. This mechanistic detail would strengthen the model and distinguish it more clearly from CD40/BAFF signaling.

<u>>>Response:</u> Thanks for these interesting suggestions, but these are unfortunately quite difficult to address experimentally. As pointed out by the reviewer, TRAF3 remains stable. We suspect that this is the case because LMP1 and TRAF3 drive rapid depletion of cIAP1 and 2, which are implicated in TRAF3 turnover in the context of CD40 signaling (PMID 18997792). To our knowledge, TRAF3 is not known to trigger its own degradation through autoubiquitination. In support, we confirmed that Daudi B cell CD40L treatment significantly decreased TRAF3 expression levels, whereas LMP1 expression did not (new Fig. S1C and below). Furthermore, CD40L induced higher levels of TRAF3 polyubiquitination than LMP1 (see below and new Fig. S5A).

We agree that it is a potentially interesting possibility that LMP1 might instead induce another type of PTM to stabilize TRAF3. However, it is quite difficult to identify such PTMs in an open-ended search. Without known what modification we are looking for such as ubiquitination, our proteomics core points out that we would need to identify a shifted band on a Coomassie blue stained gel, or at least silver stained gel, of modified TRAF3 that could be excised and analyzed. This is not possible, as we do not observe even by western blot such a shifted form of TRAF3.

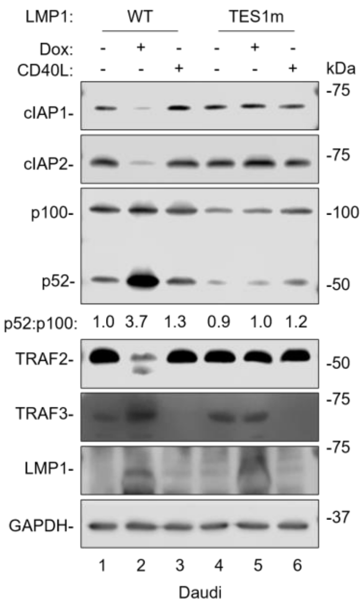

New Fig. S1C. Analysis of LMP1 and CD40L effects on cIAP1, cIAP2, TRAF2 and TRAF3 expression. Immunoblot analysis of WCL from Daudi B-cells induced for WT or TES1m LMP1 expression by addition of 250ng/mL Dox for 24 hours, or treated with 50 ng/mL CD40L for 1 hour. p52:p100 ratios are indicated.

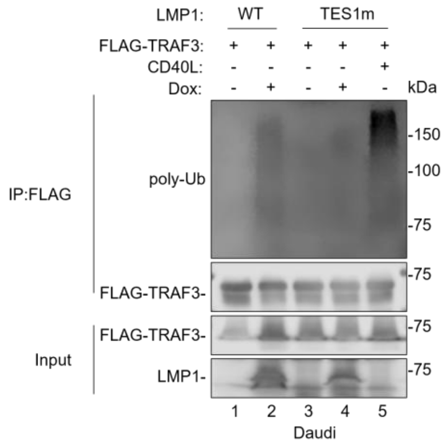

New Fig. S5A. Immunoblot analysis of input versus anti-FLAG-TRAF3, immunopurified from Daudi cells that were transfected with expression vectors encoding FLAG-tagged TRAF3. Daudi cells were induced for WT or TES1m LMP1 expression by 250 ng/mL Dox for 24 h or treated with 50 ng/mL CD40L for 1 hour. Cells were then treated with MG132 (5µM) for an additional 8 hours before collection. Cell lysates were boiled and immunoprecipitated with anti-FLAG antibody and protein A/G magnetic beads.

3. The manuscript would benefit from more quantitative analysis of protein-protein interactions. The authors should determine the stoichiometry of TRAF3:cIAP1:cIAP2 within LMP1 complexes and provide more rigorous quantification of the co-immunoprecipitation experiments. Co-immunoprecipitation experiments should include quantitative controls and potentially mass spectrometry to define complex composition more precisely. Additionally, time-course experiments comparing the kinetics of cIAP1/2 degradation versus NIK

accumulation would better establish the temporal sequence of events and strengthen the mechanistic model.

__>>Response:__ While interesting, the stoichiometry analysis is unfortunately not feasible in our opinion. LMP1 forms ladders of high molecular weight oligomers (within a cell, it is likely that LMP1 oligomers form at lipid raft signaling sites that contain a range of LMP1 copy numbers). Further complicating matters, LMP1 associates with distinct TRAF trimers, which likely include TRAF3 homotrimers, TRAF2:TRAF3 heterotrimers, TRAF3:5 heterotrimers, TRAF2:3:5 heterotrimers, etc. Adding to the complexity, LMP1 targets cIAP1/2 for degradation, making it difficult to accurately quantitate their abundance in LMP1/TRAF complexes. It would not be possible to define stoichiometry by immunoblot, as different antibodies would yield different quantitative results…one would essentially need to knock the same epitope tag onto different complex components to be able to quantitatively cross-compare abundances in IP/western experiments. We also wish to note that LMP1 has been difficult to analyze by LC/MS – in our analyses of newly infected primary B-cells, we rarely if ever observe LMP1 peptides from our mass spec data. We suspect that this relates to the high hydrophobicity of the TM regions, causing it to be retained on the C19 column that is commonly used to separate peptides by liquid chromatography prior to MS analysis, and perhaps also by the relatively few trypsin cleavage sites (for instance, LMP1 contains a single lysine residue). Trypsin is commonly used for LC/MS analyses by our core, and while we could work with them to devise different chromatography and cleavage conditions, the costs would be prohibitive.

As suggested, we performed time-course experiments. LMP1 expression was induced in Daudi cells. As shown in the new Fig. S5C and below, LMP1 induction rapidly depleted cIAP1 and cIAP2 levels by 8 hours post-Dox treatment. NIK abundance was maximally increased at the 8 hour timepoint. p100:p52 processing reached peak levels by 12 hours. These data highlight that LMP1-driven cIAP1/2 depletion precedes noncanonical NF-κB activation.

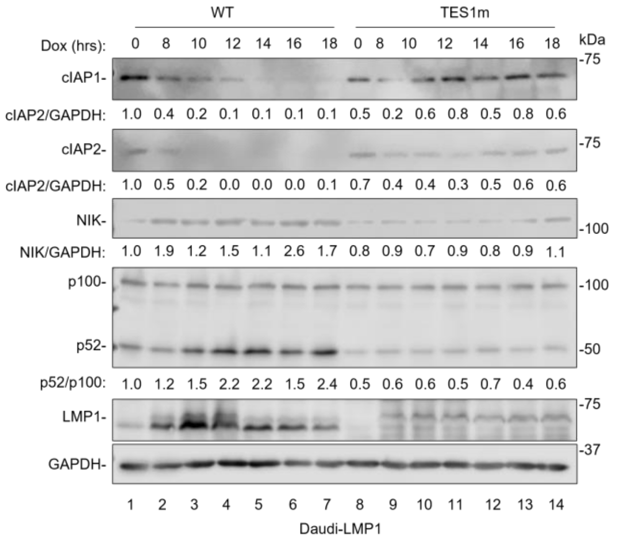

New Fig. S5C. Analysis of LMP1 on cIAP1, cIAP2 expression, and p100 to p52 processing in Daudi Burkitt cells in a time dependent manner. Immunoblot analysis of WCL from Daudi cells induced for WT or TES1m LMP1 expression by addition of Dox (250ng/mL) for the indicated hours. GAPDH- normalized densitometry or p52:p100 ratios are shown below each lane and were also normalized to WT unstimulated control ratios, which were set to 1.

Part III – Minor Issues: Editorial and Data Presentation Modifications
Please use this section for editorial suggestions as well as relatively minor modifications of existing data that would enhance clarity.

Reviewer #1: 1. It would be appreciated if the authors can address this criticism: Study LMP1 signaling and EBV type III latency using EBV+ Burkitt lymphoma (BL) cell lines as models. However, EBV+ BLs in humans do NOT express type 3 latency since they have a stringent type I latency in which LMP1 is not expressed to any degree. The BL cell lines used by the investigators are due to a cell culture artifact ("type III latency") that occurs using certain EBV BL cell lines in tissue culture; BL cell lines with type III viral latency are not thought to be good models for actual human BLs. That said, the findings are not physiological relevant to BL. It is also unclear whether LMP1 shares the same mechanism in signaling transduction in BL and other EBV+ cancers.

>>Response: We agree that Burkitt lymphoma cells typically have EBV latency I and therefore do not express LMP1. As such, the role of LMP1 described in this study is not related to typical Burkitt lymphoma biology (though we do wish to point out reports that suggest that at the single cell level there may be more LMP1 expression in Burkitt tumors than previously recognized). Instead, we used Burkitt cells solely as a B cell model system in which to study conditional LMP1 signaling, as their LMP1-null background allows direct comparison between LMP1-induced vs LMP1-uninduced cells, and allows cross-comparison between wildtype LMP1 and the TES1 point mutant. We could not simply knockout LMP1 in LCLs, because they are dependent on LMP1 for survival. Importantly, use of Burkitt cells allowed us to cross-compare WT vs TES1m LMP1, which would not be possible in LCLs. However, to ensure that the findings obtained in Burkitt cells are relevant to physiologic latency III conditions, we also performed key experiments in EBV-infected primary B cells and LCLs, which naturally express LMP1, including an LCL generated from an EBV BAC in which LMP1 was FLAG-tagged and expressed from the EBV genome at physiological levels. These experiments (Fig. 1D, 3C, 4D and 6C) yielded results consistent with those observed in the Burkitt lymphoma model.

2. Loading controls are missing in some blots where they are necessary.

>>Response: Load controls were included in all figures. We suspect that the reviewer is referring to Fig. 1D, where we used DDX1 as the loading control for our studies of EBV infected primary human B cells. Our lab has used DDX1 as a load control in multiple recent studies, as its expression remains stable between resting primary B-cells, upon EBV B-cell transformation or upon immunoreceptor stimulation (PMID: 31257153, 31518366, 34281398, 36070681). This information has been included in the figure legend. For our IP western studies, we show inputs in all figures and normalized by cell numbers. It is not customary to include GAPDH type load controls in IP/western experiments.

3. Figures are generally clear, but some blots (e.g., TRAF2 cleavage bands) would benefit from higher resolution or inclusion of molecular weight markers.

>>Response: Thanks for this suggestion. As suggested, we improved the resolution of the TRAF2 and other blots, and also provided molecular weight markers for all the blots.

4. The discussion could expand briefly on potential consequences of cIAP depletion for TNFR-mediated pro-survival signaling, as this might create vulnerabilities exploitable therapeutically.

>>Response: We thank the reviewer for this interesting suggestion. We now expand the discussion section as suggested.

5. The analogy to SMAC mimetics is intriguing, but no experiments directly test whether SMAC mimetics modulate LMP1 TES1 signaling in EBV+ B cells. Given the translational framing, inclusion of such experiments or a more cautious discussion would be appropriate.

>>Response: Thanks, we now cross compare SMAC mimetics and LMP1. To address this point, we directly cross-compared LMP1 and SMAC mimetic (SMACm) effects in Daudi cells. We chose to use Daudi cells with inducible LMP1 expression rather than LCLs, because LCLs are dependent on constitutive LMP1 expression for survival, and it would not be possible to cross-compare LMP1 with SMAC mimetic effects. Adding SMAC mimetics to LCL would combine LCL and SMAC mimetic effects, and we would therefore only be able to test superimposed LMP1 and SMACm effects, which might be difficult to interpret. As shown in new Fig. S5B and below, LMP1 expression or SMACm treatment each deplete cIAP1 and cIAP2, and each induced p100:p52 processing.

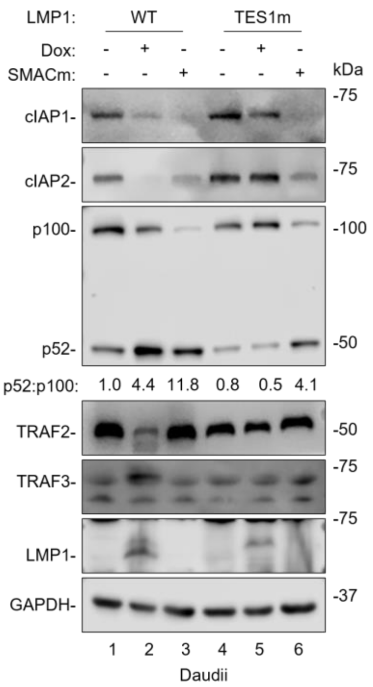

New Fig. S5B. Analysis of LMP1 and SMACm (Birinapant, SelleckChem) effects on cIAP1, cIAP2, TRAF2 and TRAF3 expression in Daudi Burkitt cells. Immunoblot analysis of WCL from Daudi cells induced for WT or TES1m LMP1 expression by addition of 250ng/mL Dox for 24 hours, or treated with 20 uM SMACm for 8 hours. p52:p100 ratios are indicated.

However, SMAC mimetic birinapant treatment did not alter TRAF2 expression or electrophoretic mobility.

Reviewer #2: Minor comments

1) The EBV latency section in the Introduction could be shortened to streamline focus on LMP1 and NF-κB signalling.

__**>>Response:**__ Thanks, we removed the description of the latency II program but retained the sections on latency I and III, as these two programs are directly compared in our study.

2) Figures 5 and 6 are dense. Consider adding schematic cartoons.

__**>>Response:**__ We thank the reviewer for this suggestion. We added schematic cartoons to Figure 5 and 6.

3) Figure legends could better highlight which results support TES1 vs TES2 dependency.

__**>>Response:**__ We revised legend for Fig 1A to clearly indicate TES1 and TES2 signaling capacities of each construct.

4) There is some redundancy between the Results and Discussion sections that could be reduced for conciseness (e.g., TES1 vs TES2 role is repeated multiple times).

__**>>Response:**__ We revised the discussion section as suggested to improve conciseness.

5) Ensure consistent usage of "TES1/2" vs "CTAR1/2" throughout.

__**>>Response:**__ We revised the manuscript to consistently use "TES1/2" throughout.

Reviewer #3: 1. Direct comparison with CD40 signaling in identical experimental systems would strengthen the claims about mechanistic distinctions. The authors could perform side-by-side experiments demonstrating how CD40 targets TRAF3 for degradation while LMP1 preserves TRAF3 but degrades cIAPs. This comparison would validate that the observed effects are truly LMP1-specific rather than only relevant in the experimental systems used. It would also be valuable to test whether other TRAF3-binding receptors can similarly recruit and activate cIAP1/2.

__**>>Response:**__ As suggested, we induced LMP1 expression in Daudi cells or treated them with CD40L. As shown in new Fig. S1C and below, LMP1 overexpression downregulated cIAP1, cIAP2, and TRAF2, but not TRAF3. BY contrast, CD40L depleted TRAF3, but had minimal effect on cIAP1, cIAP2 or TRAF2 levels.

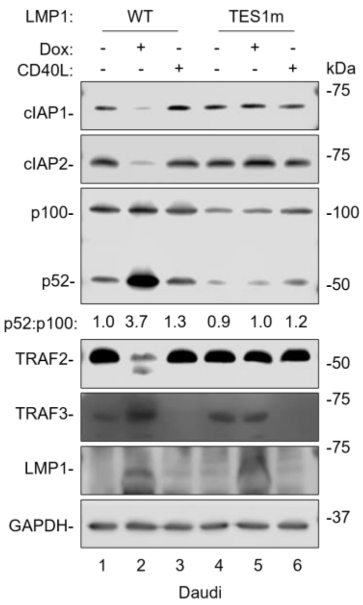

New Fig. S1C. Analysis of LMP1 and CD40L effects on cIAP1, cIAP2, TRAF2 and TRAF3 expression. Immunoblot analysis of WCL from Daudi cells induced for WT or TES1m LMP1 by addition of 250ng/mL Dox for 24 hours, or treated with 50 ng/mL CD40L for 1 hour. p52:p100 ratios are indicated.

2. The low molecular weight TRAF2 bands are intriguing and could be further explored, though a bit beyond the scope of the manuscript. What is the specific protease responsible for this cleavage and/or does this processing affect other TRAF2 functions beyond non-canonical NFκB signaling? The authors could identify the cleavage sites through N-terminal sequencing or perform complementation studies with cleavage-resistant TRAF2 mutants. Understanding TRAF2 processing may reveal additional layers of LMP1's regulatory mechanism and could be important for interpreting the functional consequences of the pathway.

<u>>>Response:</u> Please see the response to Reviewer #1 point #3 above.
